# A cost-effective and efficient reprogramming platform for large-scale production of integration-free human induced pluripotent stem cells in chemically defined culture

**DOI:** 10.1038/srep11319

**Published:** 2015-06-11

**Authors:** Jeanette Beers, Kaari L. Linask, Jane A. Chen, Lauren I. Siniscalchi, Yongshun Lin, Wei Zheng, Mahendra Rao, Guokai Chen

**Affiliations:** 1 Center for Molecular Medicine, National Heart, Lung and Blood Institute, Bethesda, Maryland; 2 National Center for Advancing Translational Sciences, National Institutes of Health, Bethesda, Maryland; 3 Center for Regenerative Medicine, National Institutes of Health, Bethesda, Maryland; 4 Faculty of Health Sciences, University of Macau, China

## Abstract

Factors limiting the adoption of iPSC technology include the cost of developing lines and the time period that it takes to characterize and bank them, particularly when integration free, feeder free, and Xeno-free components are used. In this manuscript we describe our optimization procedure that enables a single technician to make 20–40 lines at a time in a 24–96 well format in a reliable and reproducible fashion. Improvements spanned the entire workflow and included using RNA virus, reducing cytotoxicity of reagents, developing improved transfection and freezing efficiencies, modifying the manual colony picking steps, enhancing passaging efficiency and developing early criteria of success. These modifications allowed us to make more than two hundred well-characterized lines per year.

Somatic reprogramming with defined factors can convert differentiated human somatic cells into induced pluripotent stem cells (iPSCs)^1^,^2^. This iPSC technology has been used to generate thousands of donor specific iPSCs for disease modeling, drug screening and potentially cell therapy^3^. The high cost of the iPSC derivation is a limiting factor for most laboratories when multiple patient samples need to be reprogrammed. For example, among the most popular integration-free methods, Sendai virus^4^ and mRNA^5^ approaches need expensive reagents for reprogramming, while the episomal approach requires large numbers of starting cells that lead to high labor costs^6^. At the same time, reprogramming and early colony expansion usually take about two months, and the labor cost is very high, especially at the colony expansion stage when many colonies need to be selected, expanded and frozen at the same time. In addition, different iPSC colonies may emerge and expand at a different pace, so it is difficult for researchers to handle so many samples at different stages of reprogramming. For all these reasons, many researchers are limited to 2–4 reprogramming samples per person in each experimental cycle, and such methods are not efficient enough to satisfy projects that need multiple controls and multiple patient samples. For example, a six-sample iPSC project, including 3 controls and 3 patients, could cost more than $10,000 in reagents with one full-time researcher to generate iPSCs even before any characterization and clone selection.

In order to meet the increasing demand of the large-scale production of high quality iPSCs, we set out to develop an efficient integration-free reprogramming platform in a chemically defined E8 medium system. We designed the platform with the goal that one researcher could manually handle 20 to 40 patient samples while performing other routine lab duties, and the cost of the derivation per sample would be kept to a minimum. In addition, the system should be suitable for adaptation to an automated system if such equipment is available. In order to allow the platform to be applicable to the majority of potential patient samples, we added chemically defined lipid mix during reprogramming to make sure that cells with defects in metabolism or lipid function could also be reprogrammed efficiently. After considering the advantages and disadvantages of different integration-free reprogramming methods, we chose the CytoTune^TM^ Sendai virus approach, because of its high reprogramming efficiency and single transduction procedure^4^,^7^,^8^. We specifically optimized and developed new practices on four aspects (Fig. 1A and Table S1): 1. Lower reagent cost; 2. Tight control on reprogramming schedules for high throughput reprogramming; 3. Minimal but efficient cell culture handling, 4. Frequent and efficient preservation and storage of cells as a backup. Here we describe how a cost-effective and efficient reprogramming platform was built for production of a large number of integration-free iPSCs in chemically defined medium.

## Results

**Figure 1:**
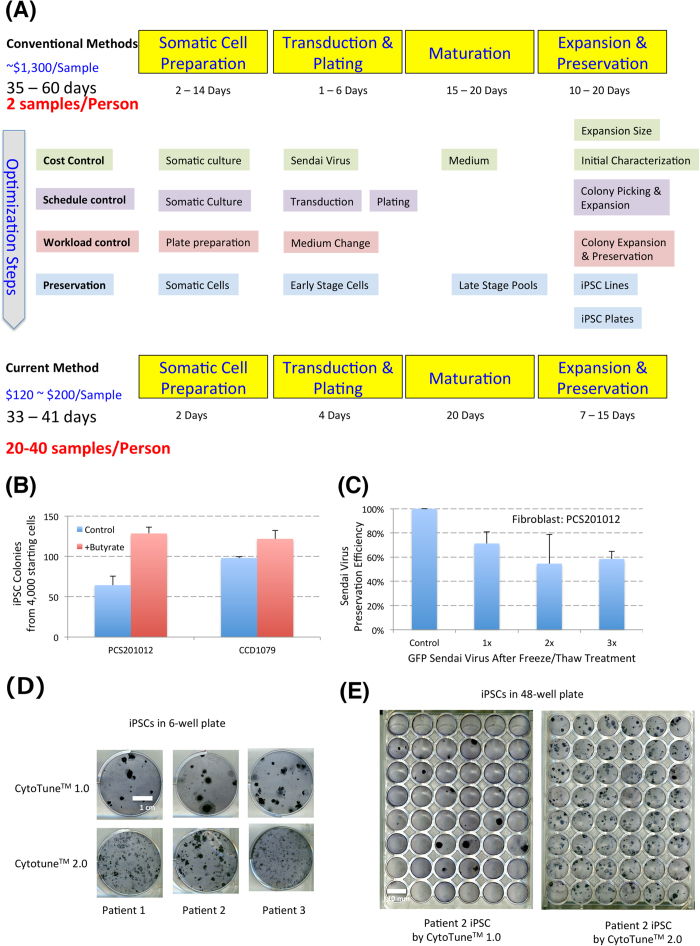
Sendai Viral Reprogramming in small scale. **A**. *Flow chart for the optimization from conventional methods to current method.* Four main aspects were improved to improve reprogramming efficiency and handling capacity in a regular lab setting. **B**. *Confirmation of the enhancement of butyrate in Sendai Viral reprogramming.* Two control fibroblast lines (ATCC: PCS201-012 and CCD1079) were reprogrammed with the CytoTune^TM^ 2.0 Sendai Viral Reprogramming kit, and then reprogrammed in medium with or without 100 uM Sodium Butyrate. The iPSC Colonies were counted after 25 days. Experiments were done in triplicate (PCS201-012, p < 0.0015; CCD1079, p < 0.023). **C**. *Viral activity conservation after freeze/thaw cycles.* GFP-control Sendai virus was thawed and then refrozen for three times, and a portion of virus was taken at each cycle to apply to fibroblasts (PCS201-012). GFP expression in the population was analyzed two days after transduction, and the transduction efficiency was normalized by virus without the freeze/thaw cycle (control). The results were confirmed in another line CCD1079 (data not shown). Experiments were done in triplicate (3x vs control, p < 0.001). **D***. Comparison of CytoTune*^*TM*^ *1.0 and CytoTune*^*TM*^ *2.0 kits.* The three patient samples were transduced by two kits respectively with MOI 3, and the cells were then plated and cultured by the same method in 6 well plate (Fig. 1A), and the iPSC cells were stained by APS staining 25 days after transduction. **E**. *Reprogrammed cells plated on Matrigel.* The cells transduced in 1D were plated in a 48-well plate, and cultured and stained by the same procedure.

First, we tried to decrease the cost of reprogramming for each sample. The high cost of Sendai virus and potential cytotoxicity have become the biggest obstacles to its use for most labs. We used the less toxic lentiviral STEMCCA approach^9^ to screen popular reprogramming enhancers in defined culture and identified butyrate as an efficient supplement to improve viral induced reprogramming in defined feeder-free culture (Figure S1A)^9^,^10^, and then incorporated the beneficial effect of butyrate to enhance Sendai Virus induced reprogramming (Fig. 1B).

Based on limited tests with two control fibroblast cell lines, one kit of Sendai Virus could potentially generate up to 60,000 individual iPSC colonies from 2 million starting cells, thus one kit can be potentially used on 100 patient samples (20,000 cells each) that could generate 600 iPSC colonies for each sample (Fig. 1B). Considering that some patient cells could have significantly lower reprogramming efficiency, we decided to use one kit to reprogram 24–48 samples as routine practice. We tested the preservation of viral activity with GFP Sendai virus control under different treatments, (Fig. 1C and Figures S1B and S1C), and found that close to 80% activity was maintained, and >50% activity was maintained even after 3 cycles of repetitive freeze and thaw processes in its original stock solution (Fig. 1C). This allows researchers to thaw Sendai virus and preserve its activity in small aliquoted frozen stocks that could be used for reprogramming. Considering the handling capacity in the expansion stage, we usually aliquot each Sendai Virus kit to 5 portions so that each aliquot could be used to reprogram 5 to 20 lines.

Sendai virus showed severe cytotoxicity especially in CytoTune^TM^ 1.0 during reprogramming, and batch testing was usually necessary. The new generation of CytoTune^TM^ 2.0 showed less cytoxocity and higher reprogramming efficiency compared to CytoTune^TM^ 1.0 (Fig. 1D,E). 1/50 of the kit is sufficient to generate iPSCs with both CytoTune^TM^ 1.0 and CytoTune^TM^ 2.0 kits. We recently started to use CytoTune^TM^ 2.0 that potentially could use far less Sendai virus for more samples per kit.

Spin transduction increased the transduction efficiency of Sendai virus (Figure S1D), and we used it as a backup strategy in the case the cells could not be effectively transduced for reprogramming. We found even without centrifugation, somatic cells were efficiently transduced mostly in the first 6 hours (Figure S1E). 1/100 of the kit was enough to generate iPSCs consistently from tested lines by CytoTune^TM^ 2.0 (Figure S1F). After decreasing the cost of virus, we also addressed the usage of reprogramming medium in the later part of the paper, which is another big cost component.

**Figure 2:**
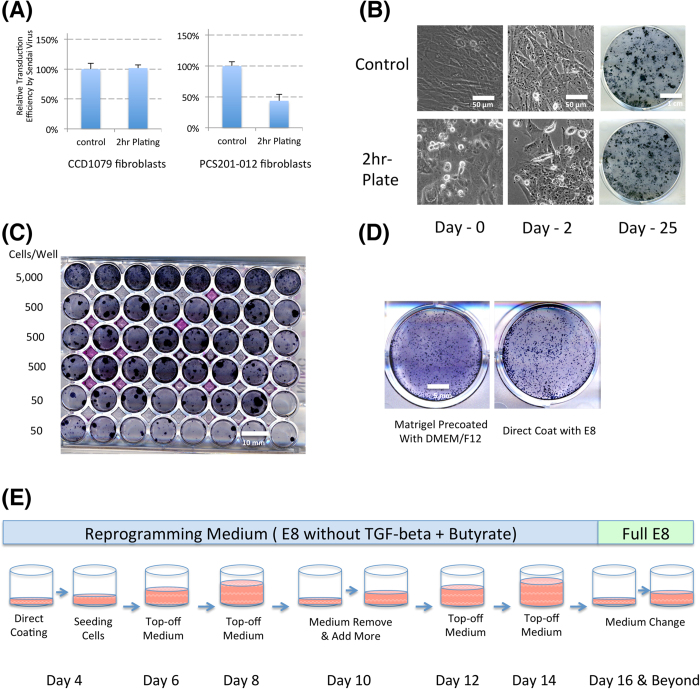
Reprogramming timing and workload control. **A**. *Viral transduction in cells at different plating time points.* Fibroblast cells (PCS201-012 right graph and CCD-1079 left graph) were cultured for 2 days to confluence, and one set of cells were directly transduced with GFP sendai virus, and the other set of cells were dissociated and then replated 2 hours before the transduction, and the GFP expression was analyzed 2 days after the transduction. Experiments were done in triplicate (CCD1079, p > 0.30; PCS201-012, p < 0.05) **B**. *Comparison of reprogramming efficiency for cells with different plating timings.* Fibroblast cells were cultured for 2 days until confluence, and one set of cells were directly transduced with CytoTune^TM^ 2.0 virus, and the other set of cells were dissociated and then replated 2 hours before the transduction, and the colonies were stained 25 days after transduction. **C**. *Individual iPSC colony emergence after serial dilution.* **D***. Cell survival on plates coated directly with E8 medium.* E. *Medium change schedule during reprogramming.*

Second, the reprogramming schedule of multiple samples was tightly controlled to make sure a researcher could be allocated with more uniform and synchronized duties at each stage. One of the most unanticipated difficulties for reprogramming is to gather enough patient samples simultaneously to start the experiments. One big advantage of Sendai viral reprogramming approach is that it needs around 1 × 10^4^ starting cells, far fewer than other methods. This shortens the reprogramming by an average of at least 2 weeks. More than 20 individual reprogramming experiments could be initiated simultaneously just one day after thawing. In order to closely synchronize the reprogramming process, freshly passaged cells were transduced and reprogrammed around 2 hours after plating (Fig. 2A), so researchers could adjust the starting cell density of many lines, and start the reprogramming quickly and simultaneously. The transduced cells often showed significant morphological changes two days after successful transduction (Fig. 2B), which allow people to quickly evaluate whether the viral transduction for reprogramming is efficient. If the transduction is not successful, the repeat experiments could be performed two days after the initial experiment.

The experimental timing can also be affected at the colony expansion stage. If picked colonies are directly picked into individual wells, the receiving plates are consistently exposed to potential contamination, so we usually picked the colonies into E8 medium with ROCK inhibitors in individual vials before all cells are pipetted and finally transferred into wells. This significantly decreased the potential of contamination and ensured the smaller pieces when plating. Alternatively, reprogrammed cells could be plated directly onto a 48-well plate after the first split for clonal reprogramming and expansion (Figs 1E and 2C). Given that the reprogramming efficiency for different patient lines can easily vary by 100-fold, doing a serial dilution down the 48 well plate ensures that all reprogrammed lines should have a single colony/well in one of the bottom three rows, or even a higher row if the reprogramming efficiency is low. In nearly all cases, we found that the top row of cells on the 48 well plate had 5–50 colonies/well. This method allows for the development of a single colony/well and the passaging of this colony by EDTA/PBS with ROCK inhibitors rather than manual picking. These modifications effectively allow most iPSC colonies to be expanded at similar time frames. With the above modifications, most patient cells follow a very similar reprogramming timing, and are ready for clonal expansion at the same time.

Third, we decreased the cell culture workload to allow a large number of iPSCs to be handled by limited manpower especially during the colony expansion stage. Cell culture medium is a major cost component, and every medium change is also labor intensive and exposes the samples to potential contamination. To save medium cost and labor, we directly coat plates with Matrigel or other coating materials in reprogramming medium or E8 medium without a medium change before seeding, utilizing the fact that the medium has a low protein content (lack of albumin) and does not block the coating (Fig. 2D). This practice saves one round of medium change at each passage. At the same time, we followed a 2-day feeding schedule to add fresh medium, and removed spent medium every 6 days (Fig. 2E) until day 16 or when cells reached around 30% confluence and medium started to turn yellow. This practice could potentially save 70% of medium during reprogramming at already downsized scale.

**Figure 3:**
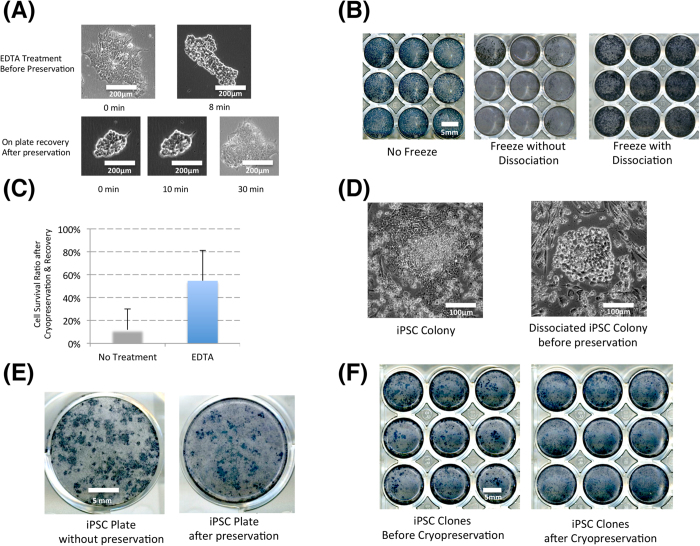
Cryopreservation in Reprogramming. **A**. *Human ESC dissociated before preservation on the plate.* H1 hESCs were dissociated on plate before cryopreservation. **B**. *H1 ESC recovery after cryopreservation on plate.* Three identical sets of H1 ESCs were prepared on 48 well-plates. One set was directly stained with APS staining, the other two sets were cryopreserved either with or without dissociation, and cells were then recovered in E8 and later stained by APS staining kit. **C**. *Cryproservation efficiency on plate.* The cells from 3B were counted by flow cytometry, 6 untreated and 6 EDTA-treated samples (p < 0.01) **D***. iPSC dissociation for cryopreservation.* iPSC reprogramming plates were treated with EDTA/PBS before cryopreservation. **E**. *On-plate preservation of iPSC Colonies.* Original iPSC reprogramming plates were directly preserved and recovered after brief EDTA dissociation. **F**. *Cryopreservation of expanded iPSC colonies.* Two sets of iPSC clones were cryopreserved two days after in-well dissociation.

**Figure 4:**
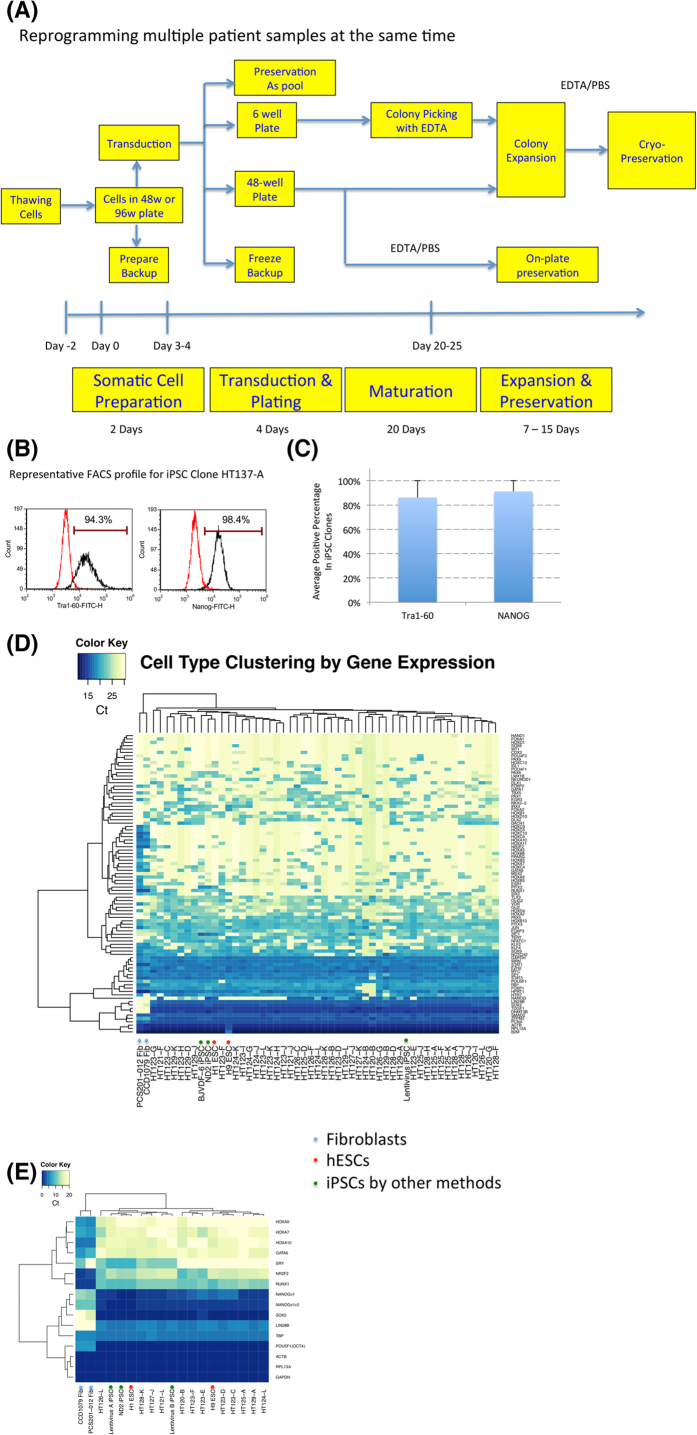
Cost-effective and efficient reprogramming platform and characterization. **A***. Flow chart for reprogramming of multiple lines*. **B**. *Typical flow cytometry profile of iPSCs.* iPSCs were expanded for two passages after reprogramming and stained with Tra1–60 and NANOG antibodies with fluorescent markers. **C**. *Average staining from 189 iPSC clones (derived from 72 cell lines).* 189 iPSC clones were clonally expanded and stained, and the average NANOG/Tra1–60 positive ratios were shown **D**. and **E**. *Pluripotent and differentiation gene expression profile in qPCR analysis and Fluidigm Biomarker qPCR analysis.*

In addition, we also used the EDTA/PBS method to passage iPSCs, which needs no neutralization and centrifugation. This also saves more than half of the work needed during each passage. Because of the iPSC-enriching feature of EDTA/PBS treatment, the iPSCs are usually ready to be frozen or analyzed in two passages, and the expanded iPSC colonies were usually high in purity without additional selection steps (Figs 3 and 4).

Fourth, we developed multiple steps for backup and preservation during the reprogramming process. Because of the large amount of patient samples involved in the process, it is important to safeguard the resources invested in each round of the experiment at each step, from the preparation of parental cells to clonal expansion. Before the viral transduction, one or two duplicate plates of parental cells are prepared to quickly repeat experiments if there is any failure. A portion of cells was frozen in the first split after reprogramming as an initial backup pool, and these cells can be thawed out in case additional experiments need to be performed (Figure S2A). When the reprogramming experiment reaches around 20 days, one or two wells of reprogrammed cells could also be cryopreserved as backup pools.

During colony expansion, cells are frequently preserved as stock starting from Passage 3. When more than 20 patient samples and more than 100 iPSC colonies are involved, the expansion and cryopreservation becomes an overwhelming task even for the most experienced researcher. EDTA/PBS dissociated iPSCs could routinely be efficiently cryopreserved in vials without neutralization and centrifugation^11^, but it is still very labor-intensive to preserve cells in individual vials and thaw them afterwards. To address this issue, in Fig. 3A–C, we show that the dissociated ESCs could be efficiently cryopreserved on plates directly. Such treatment could also be applied to iPSC colonies and lead to the preservation of iPSCs in cell culture wells on multi-well plates (Fig. 3D–F). This method greatly expanded the capacity to preserve large numbers of iPSC clones. It is not only helpful for the picked and individually expanded iPSC colonies, but also greatly helped the reprogramming experiments conducted in the 48 or 96 well format. Traditionally, only 3 to 5 colonies are expanded for preservation, and the new procedure allows hundreds of lines to be preserved with minimal effort for short-term backup (Fig. 3D). In randomly tested iPSCs frozen with the new method, most cells maintained normal karyotypes, and the abnormal iPSC lines might be from defective parental lines or the reprogramming process (Figure S2C and Table S2). We foresee that this procedure can also be very helpful for gene targeting experiments that need to handle and preserve many lines in the same set.

With all the improvements at each step of the process, we are able to generate hundreds of iPSC lines with minimal resources, and control the reprogramming experiments in a more confined time frame (Fig. 4A). More than 800 iPSC lines have been established from more than 160 lines with this platform, and the expanded colonies demonstrated high purity by pluripotency markers in flow cytometry analysis (Fig. 4B,C). We also used the Qiagen RT2 Profiler PCR Array of Human Stem Cell Transcription Factors in the Fluidigm format to quickly evaluate pluripotent gene expression in 48 to 96 iPSC lines compared to somatic cells, and iPSCs derived in the new platform showed signature pluripotency marker expression (Fig. 4D). We were also able to use a smaller primer-set to quickly analyze iPSC quality with satisfactory results (Fig. 4E).

By increasing efficiency and consistency, we decreased the experimental scale of individual reprogramming by up to 50 fold (Figure S1E), and increased the number of reprogramed patient samples by 10 fold, while decreasing the medium usage by 70%, and thus saved the reagent cost by more than 80% per patient sample (Supplemental Tables 5 and 6). Using EDTA/PBS for passaging and cryopreservation and coating plates directly with Matrigel in culture medium generally cut the cell culture workload by half.

## Discussion

We successfully improved four aspects of reprogramming including lower reagent cost, tight control on reprogramming schedules, minimal but efficient cell culture handling, and efficient preservation and storage of cells as a backup. The synchronized reprogramming allowed researchers to focus on each stage’s needs without dealing with cells at different stages that require different treatments and media. The new cryopreservation procedure greatly enhanced the capacity to handle many lines. With our new platform, we have drastically decreased the cost and time involved in reprogramming, which is becoming more and more important as the demand for new iPS lines increases. Our method makes it possible for even a small lab to efficiently produce these iPS lines. In summary, this optimized reprogramming platform can help generate large number of iPSC clones in regular lab setting and will be a very useful method for the stem cell field.

## Methods

Unless otherwise specified, all statistical data is the result of 3 independent experimental repeats, in which numbers were collected independently at the conclusion of the experiment. Alkaline phosphatase staining is a common way to estimate reprogramming efficiency, and it was used during the protocol development stage as presented in Fig. 1 and Figure S1. However in practice, we find that many APS positive colonies do not survive passaging or differentiate within a few passages. In our reprogramming experiments, the success was rated by following criteria: 1. Colonies were observed with iPSC morphologies; 2. Cells were successfully picked and expanded in E8 medium while maintaining stem cell morphologies; 3. Expanded cells were stained with positive OCT4 and Tra1–60 expression; 4. Selected lines were further tested by qPCR for more stem cell markers (Fig. 4D). Additional assays were done by collaborators (end users) who tested the pluripotency by embryoid body differentiation or/and teratoma formation. Usually a maximum of 12 colonies were picked to expand between 3–6 clones.

### ESC/IPSC culture

ESC: H1 cells, WA01 (US National Institutes of Health (NIH), human ESC registry no. 0043). ESC/iPSC cultures were maintained on Matrigel (Corning 354230) in the chemically-defined Essential 8 medium (Life Technologies A1517001) and passaged by the EDTA method, as described previously^11^.

The experimental designs using human ESCs and iPSCs were approved by the Institutional Biosafety Review committee in the National Institutes of Health. The Use of anonymous human samples for reprogramming was approved by the Institutional Review Board.

### Fibroblast Cell Culture

Human fibroblast lines PCS201-012 (PCS-201-012) and CCD-1079Sk (CRL-2097) were obtained from the ATCC, and cultured in DMEM high-glucose media (Life Technologies 11195-073) containing 10% fetal bovine serum, penicillin/streptomycin (Life technologies 10378-016), non-essential amino acids (Life Technologies 11140-050) and L-glutamine (2 mM) until several days into reprogramming.

### Reprogramming

For reprogramming GFP Sendai virus, Cytotune 1 (A1378001) and Cytotune 2 (A16517) Sendai virus kits were purchased from Life Technologies. In the case of the GFP Sendai virus, cells were infected according to the kit instructions, and cells were maintained in the DMEM culture medium above until they were analyzed 2 days after transfection.

Fibroblasts were grown to confluence in 1 well of a 12 well plate. On day 0 or 1, they were passaged and the entire well of cells was plated into 1 well each of two 48 well plates. One plate was used for reprogramming, while another can be used as backup.

For the Cytotune 1 and 2 kits for reprogramming, cells were infected according to kit instructions and grown in DMEM culture media until day 4, when they were plated onto Matrigel plates directly in Reprogramming Media 2 as described^11^ (Reprogramming Media 1 was not used in this method) and cultured until 25 days after reprogramming. Cells were switched to Essential 8 media at day 20 after infection. For plating in a 48 well plate in order to obtain single colonies per well, cells were titered at decreasing cell number down the plate, so that at least one row should have 1 colony/well. If there were many colonies per well at even the lowest plating density, wells could be passaged with EDTA during the reprogramming process and plated at a much lower density in a new 48 well plate in order to obtain single colonies. Colonies were selected for expansion based on morphology, and expanded and cryopreserved by passage 3. A sample was then harvested for FACS analysis of Tra1–60 and Nanog expression, and all clones studied were positive for these markers (representative sample, Fig. 4B,C).

### Cryopreservation of iPS reprogramming on 48 well plates

We removed media from the plate and added PBS/EDTA to the well one time (no washing). After 5 minutes at room temperature, the PBS/EDTA was aspirated and cells were resuspended in 150 ul of cryopreservation media (E8+ROCK inhibitor +10% DMSO). The plates incubated on ice for 5 minutes, then were placed between 2 pieces of Styrofoam and into a larger Styrofoam box, and transferred to a −80 freezer to store.

To thaw these cells, we removed the plate from −80 and place on top of a 37 ^o^C heat plate (if available). 750 ul of warmed E8+ROCKi were added to each well and the plate was transferred to a 37 ^o^C incubator. After 30 minutes, we checked for cell attachment on plate and removed the media, replacing it with 500 ul of fresh warmed E8+ROCKi and returned plates to the 37 ^o^C incubator.

### APS staining

APS staining was done using the BCIP/NBT alkaline phosphatase substrate kit IV (Vector Laboratories SK-5400) at day 25 after reprogramming.

### FACS

For cell counts and GFP analysis of the GFP Sendai infected fibroblasts, iPS cells were harvested from the plate using TryPLE (Life Technologies 12563-029) and the reaction stopped with DMEM media containing 10% FBS. Countbrite beads (Life Technologies C36950) were added and cells were analyzed by FACS, with cell numbers being normalized by the beads.

For reprogramming, iPS clones were analyzed by harvesting cells with TryPLE and the stopping the reaction with DMEM media containing 10% FBS. Cells were then fixed by a 10 minute incubation at room temperature in 4% paraformaldehyde and then washed with PBS. Prior to FACS analysis, cells were permeabilized with 0.2% Tween-20 in PBS for 10 min at room temperature and stained with anti-Tra1–60-FITC (Millipore FCMAB115F, concentration as recommended by company) and anti-Nanog-Alexa Fluor 488 (Millipore FCABS352A4, concentration as recommended by company). Nonimmune controls were used at 0.5 ul per 50 ul reaction, mouse-IgG2b-FITC (Millipore MABC006F) and rabbit IgG isotype-AlexaFluor 488 conjugate (Cell Signaling 4340S).

### Gene Expression analysis

RNA was purified using TRI Reagent® Solution according to Ambion’s protocol (Life Technologies AM9738). Residual DNA was removed using the TURBO DNA-free™ kit (Life Technologies AM1907). Reverse transcription was carried out with Maxima H Minus Reverse Transcriptase (Thermo Scientific EP0751) primed with Poly N15-mer (Eurofins SP180-1) with the recommended protocol. Prior to PCR, RNA template was removed with addition of Ribonuclease H, from E. coli (Life Technologies AM2292). A 96 × 96 Dynamic Array chip (Fluidigm BMK-M-96.96) was run with Stem Cell Transcription Factors PCR Array primers (Qiagen PAHS-501Z), using the Fuidigm protocol for Fast Gene Expression Analysis using SsoFast EvaGreen Supermix with Low ROX (BIO-RAD 172-5210) on the BioMark System. Analyses and plots for RTPCR were generated using R 3.1.1 and Bioconductor’s HTqPCR packages^12^,^13^. Ct normalization was done with the normalizeCtData function using options norm.rankinvariant, pseudo.median, and Ct.max value of 35. The heatmap was generated using the plotCtHeatmap function with euclidean distance clustering dendrograms.

## Additional Information

**How to cite this article**: Beers, J. *et al.* A cost-effective and efficient reprogramming platform for large-scale production of integration-free human induced pluripotent stem cells in chemically defined culture. *Sci. Rep.* **5**, 11319; doi: 10.1038/srep11319 (2015).

## Supplementary Material

Supplementary Information

## References

[b1] Takahashi K. *et al.* Induction of pluripotent stem cells from adult human fibroblasts by defined factors. Cell 131, 861–872, 10.1016/J.Cell.2007.11.019 (2007). 18035408

[b2] Yu J. *et al.* Induced pluripotent stem cell lines derived from human somatic cells. Science 318, 1917–1920, 10.1126/science.1151526 (2007). 18029452

[b3] Takahashi K. & Yamanaka S. Induced pluripotent stem cells in medicine and biology. Development 140, 2457–2461, 10.1242/Dev.092551 (2013). 23715538

[b4] Fusaki N., Ban H., Nishiyama A., Saeki K. & Hasegawa M. Efficient induction of transgene-free human pluripotent stem cells using a vector based on Sendai virus, an RNA virus that does not integrate into the host genome. P Jpn Acad B-Phys 85, 348–362, 10.2183/Pjab.85.348 (2009). PMC3621571 19838014

[b5] Warren L. *et al.* Highly Efficient Reprogramming to Pluripotency and Directed Differentiation of Human Cells with Synthetic Modified mRNA. Cell Stem Cell 7, 618–630, 10.1016/J.Stem.2010.08.012 (2010). 20888316 PMC3656821

[b6] Yu J. Y. *et al.* Human Induced Pluripotent Stem Cells Free of Vector and Transgene Sequences. Science 324, 797–801, 10.1126/Science.1172482 (2009). 19325077 PMC2758053

[b7] Burridge P. W. *et al.* Chemically defined generation of human cardiomyocytes. Nat Methods 11, 855–860, 10.1038/Nmeth.2999 (2014). 24930130 PMC4169698

[b8] Lieu P. T., Fontes A., Vemuri M. C. & Macarthur C. C. Generation of induced pluripotent stem cells with CytoTune, a non-integrating Sendai virus. Methods Mol Bio 997, 45–56, 10.1007/978-1-62703-348-0_5 (2013). 23546747

[b9] Somers A. *et al.* Generation of Transgene-Free Lung Disease-Specific Human Induced Pluripotent Stem Cells Using a Single Excisable Lentiviral Stem Cell Cassette. Stem Cells 28, 1728–1740, 10.1002/Stem.495 (2010). 20715179 PMC3422663

[b10] Li W., Li K., Wei W. & Ding S. Chemical approaches to stem cell biology and therapeutics. Cell Stem Cell 13, 270–283, 10.1016/j.stem.2013.08.002 (2013). 24012368 PMC3898630

[b11] Beers J. *et al.* Passaging and colony expansion of human pluripotent stem cells by enzyme-free dissociation in chemically defined culture conditions. Nat Protoc 7, 2029–2040, 10.1038/Nprot.2012.130 (2012). 23099485 PMC3571618

[b12] Dvinge H. & Bertone P. HTqPCR: high-throughput analysis and visualization of quantitative real-time PCR data in R. Bioinformatics 25, 3325–3326, 10.1093/bioinformatics/btp578 (2009). 19808880 PMC2788924

[b13] Gentleman R. C. *et al.* Bioconductor: open software development for computational biology and bioinformatics. Genome Biol 5, R80, 10.1186/gb-2004-5-10-r80 (2004). 15461798 PMC545600

