# Supplementary material for: A cost-effective and efficient reprogramming platform for large-scale production of integration-free human induced pluripotent stem cells in chemically defined culture
Source: Sci Rep. 2015 Jun 11;5:11319. doi: 10.1038/srep11319 (PMC4464084; doi:10.1038/srep11319)
Supplement: Supplementary Information [file srep11319-s1.doc]

A cost-effective and efficient reprogramming platform for large-scale production of integration-free human induced pluripotent stem cells in chemically defined culture

Jeanette Beers^1^, Kaari L. Linask^1^, Jane A. Chen^1^, Lauren I. Siniscalchi^1^, Yongshun Lin^1^, Wei Zheng^2^, Mahendra Rao^3^, Guokai Chen^1,4^

**Supplementary Information**

**Table S1.** Consideration and approaches to improve reprogramming platform.

|  | **Target Procedures** | **Solutions** |
| --- | --- | --- |
| **Strategy** | Defined conditions | E8 based culture |
|  | Avoid potential complication from lipid related issues in some patient samples | Add Chemically Defined Lipid Mix to medium |
|  | Integration Free Derivation | Sendai Virus, Episomal, mRNA |
|  | Minimal Induction Procedure | Sendai Virus, Episomal |
|  | Minimal Starting Cells | Sendai Virus |
| **Initiation** | Minimal Reprogramming Reagents | 1. Improve Reprogramming Efficiency (Butyrate) 2. Preserve and use virus in small aliquot |
|  | Improve experimental consistency | 1. Batch Test for homemade medium 2. Use the same lot of commercial medium |
|  | Prepare multiple parental cells for reprogramming at the same time | Culture parental cells on 12- or 6-well plate, and seed 48- or 96-well reprogramming plate 2-3 hours before transduction. |
|  | Control cytotoxicity of Sendai Virus | 1. Perform batch test of the virus 2. Use CytoTune^TM^ 2.0 instead CytoTune^TM^ 1.0 3. Titrate viral infection MOI |
|  | Prepare for repeating experiments | Maintain all the parental cells for at least additional 2 weeks after first transduction |
| **Reprograming**  **& Maturation** | Limit culture handling in plating | Dilute coating materials with reprogramming medium to coat the plate |
|  | Avoid centrifugation in splitting after induction | 1. Use mild enzyme such as TryPLE. 2. Neutralizing enzyme activity by dilution and addition of albumin (optional). |
|  | Avoid base medium removal after coating in reprogramming | Coat the plates directly with ECM materials in reprogramming medium |
|  | Avoid overcrowdings or low density | 1. Cell plating at different density 2. Starting at small scale |
|  | Save backup cells after induction | Cryopreserve part of cells when splitting |
|  | Quick evaluation to decide whether to redo or re-plate the cells | E8 based feeder free culture |
|  | Minimal Medium Usage | Feed every other day before cells turn 50% confluence |
|  | Avoid potential complication of genetic defects in parental lines | Add Chemically Defined Lipid Mix |
| **Clonal Expansion** | Increase speed and avoid contamination during colony picking | Pick colonies first into medium or EDTA/PBS with ROCK inhibitor in individual tubes |
|  | More synchronized passaging after colony picking | EDTA/PBS Treatment of the picked colonies with ROCK inhibitors |
|  | Clonal reprogramming and expansion without picking | Direct reprogramming in 48/96 well format after initial induction and passage |
|  | Ease of medium change with manual multichannel pipette | Adjustable multichannel pipette: 48-well plate  Regular multichannel pipette: 96-well plate |
|  | Ease of colony observation | 48-well plate |
|  | Avoid base medium removal after coating in expansion | Dilute coating materials with E8 medium to coat the plate |
|  | Enrich iPSC without picking or antibody based sorting | EDTA/PBS passaging |
|  | Cell expansion without centrifugation or enzyme neutralization | EDTA/PBS passaging |
|  | Maximize the usage of used plates | EDTA/PBS passaging |
|  | High survival during passaging | EDTA/PBS passaging with ROCK inhibitor |
| **Preservation** | Efficient cryopreservation | EDTA/PBS harvest |
|  | Cryopreservation without centrifugation or enzyme neutralization | EDTA/PBS harvest |
|  | Efficient whole plate cryopreservation for backup | EDTA/PBS colony disintegration |
|  | Efficient whole plate thawing after cryopreservation | Direct thawing by warm E8 medium with ROCK inhibitor |
|  | Efficient thawing without centrifugation | EDTA/PBS Harvest  Direct re-suspend and plate thawed cells, and change medium after 0.5 – 1 hour. |
| **Validation** | Quick evaluation of iPSC colonies by a few markers | Cell to cDNA and RT qPCR |
|  | Quick evaluation of iPSC colonies by PCR array | Cell to cDNA and BioMarker with stem cell array |
|  | Immunostaining | Flow or Imaging |

**Table S2. Summary of** Karyotype Analysis after on-plate Cryopreservation

| Karyotypes of iPSCs | Tests done | Ratio |
| --- | --- | --- |
| Normal Karyotypes | 9 | 75% |
| Abnormal Karyotypes from normal fibroblasts | 2 | 16.7% |
| Abnromal Karyotypes from abnormal fibroblasts | 1 | 8.3% |

**Table S3. Individual Karyotyping results after** on-plate Cryopreservation and recovery

| iPSC Clone | Karyotypes | Note |
| --- | --- | --- |
| HT153-A | 46,XY,t(8;20)(q11.2;p13)[6]/46,XY[13] | Normal Karyotypes were observed in another clone HT153-B in this table |
| HT152-F | 46,XY,der(1)ins(1;1)(q32;q42q23)[10]/46,XY[10] | Extra copies of 1q segments are recurrent acquired abnormalities in human pluripotent stem cell cultures. |
| HT131-A | Chr 2/5; Chr 8/15 | Abnormal Parent Line |
| HT131-B | Chr 2/5; Chr 8/15 | Abnormal Parent Line |
| HT148-D | Normal | Normal |
| HT151-C | Normal | Normal |
| HT149-F | Normal | Normal |
| HT148-A | Normal | Normal |
| HT144-A | Normal | Normal |
| HT147-D | Normal | Normal |
| HT144-B | Normal | Normal |
| HT151-A | Normal | Normal |
| HT153-B | Normal | Normal |

**Table S**4. Primers used in RT-qPCR.

| Gene Name | Variant | Class | Forward | Reverse |
| --- | --- | --- | --- | --- |
| ACTB-2 |  | Reference | AAAAGCCACCCCACTTCTCT | GACCAAAAGCCTTCATACATCTCA |
| GAPDH-3 |  | Reference | GGGAGCCAAAAGGGTCATCA | TGATGGCATGGACTGTGGTC |
| GATA6-2 |  | Differentiation | GCTAGACGTCAGCTTGGAGC | CTGGAAAGGCTCTGGAGTCG |
| HOXA10-1 |  | Differentiation | CCCTTCCGAGAGCAGCAAA | TGTCTGGTGCTTCGTGTAGG |
| HOXA7-1 |  | Differentiation | TGGTGTAAATCTGGGGGTGTAA | TCGGCTCGGCATTTTGGAA |
| HOXA9-1 |  | Differentiation | GCCTTCTCTGAAAACAATGCTG | ACCAGATCTTGACCTGCCTCT |
| LIN28B-1 |  | Pluripotency | GCACATTAGACCATGCGAGCTA | CCTCAGCTCCAAACTCGTGAG |
| NANOG-3 | v1v2 | Pluripotency | TGTGATTTGTGGGCCTGAAG | AAGTGGGTTGTTTGCCTTTGG |
| NANOG-5 | v1 | Pluripotency | GGCCGAAGAATAGCAATGGT | TGGTGGTAGGAAGAGTAAAGGC |
| NR2F2-1 |  | Differentiation | GGCCATAGTCCTGTTCACCT | GAATCTCGTCGGCTGGTTGG |
| POU5F1(OCT4)-2 |  | Pluripotency | AGCGAACCAGTATCGAGAACC | CTGATCTGCTGCAGTGTGGGT |
| RPL13A-2 |  | Reference | AAGAAAAAGCGGATGGTGGTTC | ACTTCCAGCCAACCTCGTGA |
| RUNX1-1 |  | Differentiation | TACCGCAGCCATGAAGAACC | TTGCGGTGGGTTTGTGAAGA |
| SOX2-1 |  | Pluripotency | GGGGAAAGTAGTTTGCTGCC | CGCCGCCGATGATTGTTATT |
| TBP-1 |  | Reference | TGAGTTGCTCATACCGTGCTGCTA | CCCTCAAACCAACTTGTCAACAGC |

**Table S5. Success Rate of the Reprogramming Platform***

| Samples Processed | Successful in First Trial | Successful in two Trials | Failed in 3 trials | Success Rate  in first trial | Success Rate  in two trials |
| --- | --- | --- | --- | --- | --- |
| 160 | 148 | 10 | 2 | 92.5% | >98.8% |

*Failure is when trial resulted in no visible iPS colonies. Success is when 3 or more successful iPS clonal lines were developed and expanded.

**Table S6**. Estimation of the reprogramming cost in new platform*

|  | Conventional Method  2 samples by One CytoTune^TM^ 2.0 Kit | | 20-24 Samples by one CytoTune^TM^ 2.0 Kit | | 100 Samples by one CytoTune^TM^ 2.0 Kit | |
| --- | --- | --- | --- | --- | --- | --- |
|  | Size | Price | Size | Price | Size | Price |
| CytoTune^TM^ Kit | 1/2 | $1000 | 1/20 | $100 | 1/100 | $20 |
| Starting Cells | 1-2x10^6^ |  | 2 x10^4^ |  | 4 x10^3^ |  |
| Frozen Stock of transduced samples |  |  | Half of the cells on day 4-5 |  | N/A |  |
| Plates for Derivation | 3 10-cm  plates |  | One 48-well plate & 2 wells of 6-well Plate |  | One 48-well  & 1 well of 6-well Plate |  |
| Medium for Reprogramming | 300 | $120 | 100 ml | $40 | $100ml | $40 |
| Plates for Initial Passages and Freeze  (6 clones) | 9 plates |  | 3 plates |  | 3 plates |  |
| Medium for Expansion | 450 | $180 | 150 ml | $60 | 150 ml | $60 |
| Total Cost |  | $1300 |  | $200 |  | $120 |

* Note: Most of the experiments in this project were conducted with the second approach (1 kit for 24 samples), while the third approach was successful (Figure S1F), and is considered for more high-throughput or automatic systems.

**
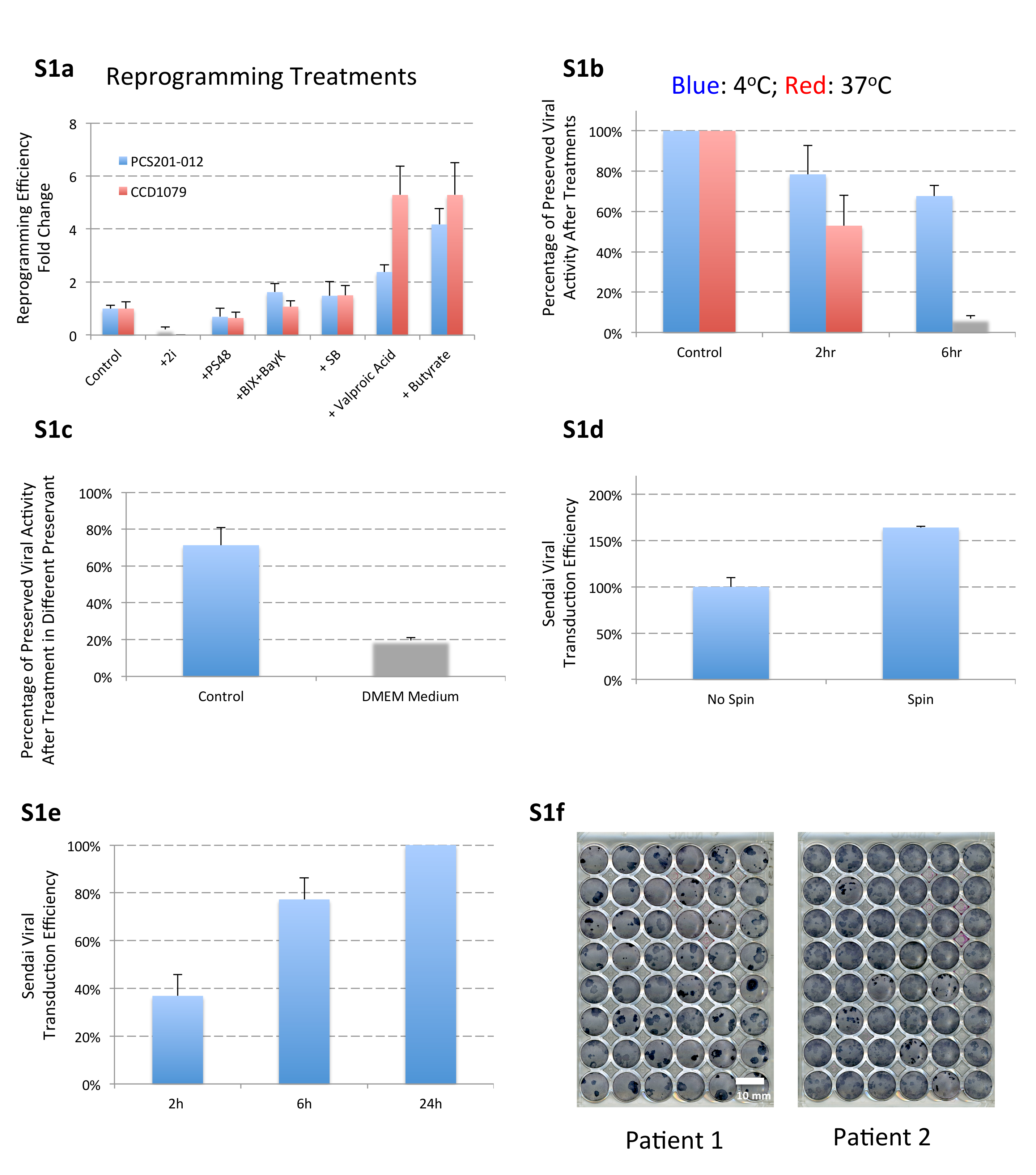
**

**Figure S1. Improvement of Reprogramming Efficiency and Condition test for Sendai Virus Handling.**

S1A. *Screening for efficient reprogramming enhancers by Lentivirus in E8 based medium system.* Two control fibroblast lines (ATCC: PCS201-012 and CCD1079) were reprogrammed with the STEMCCA lentivirus in medium containing different enhancer combinations. The iPSC colonies were counted at 25 days post-transduction. 2i - PD0325901(1μM) + CHIR99021(5μM); PS48 - Stemolecule™ PS48 (5μM); BIX+BayK – BIX01294(1μM) + R(+)BayK 8644(2μM); SB – SB431542 (10μM); Valpric Acid – Valproic Acid(5μM); Butyrate – Sodium Butyrate (100μM). Experiments were done in triplicate and p<0.017 for both lines. S1B. *Activity loss in treatments at 4^o^C and 37^o^C.* GFP-control Sendai virus was thawed and then incubated at different temperatures, and aliquots of virus were taken at each time point to infect fibroblasts (CCD1079). The GFP expression in the population was analyzed two days post-transduction, and the transduction efficiency was normalized by virus that had not undergone temperature treatments (control). S1C. *Activity loss comparison in different freezing conditions.* GFP-control Sendai virus was thawed, then diluted with DMEM/F12 dilution or not diluted, and the virus was then refrozen at -80^o^C, thawed again, and applied to fibroblasts (CCD1079). GFP expression in the population was analyzed two days post-transduction, and the transduction efficiency was normalized by virus without the dilution (control). Experiments were done in triplicate (p<0.006) S1D. *The spin protocol improves transduction efficiency.* GFP-control Sendai virus was applied to fibroblasts, and one set was spun for 40 minutes while the other was not. The GFP expression was analyzed by FACS 2 days after transduction, and normalized by the results from the set that had not been spun. S1E. *The fibroblasts were transduced by GFP-control Sendai virus, and aliquots of the supernatant were removed at different time points.*  GFP expression was scored 2 days after transduction. Experiments were done in triplicate (24 hr vs 2hr, p<0.001). S1F. *Sendai viral transduction in 96-well and then reprogrammed in 48-well format*. Fibroblasts were plated in one well of 96-well plate, were then transduced by CytoTune^TM^ 2.0 Sendai Virus, then plated in E8 based reprogramming medium with sodium butyrate on a 48-well plate, and the plate was stained by APS staining after 25 days.

**
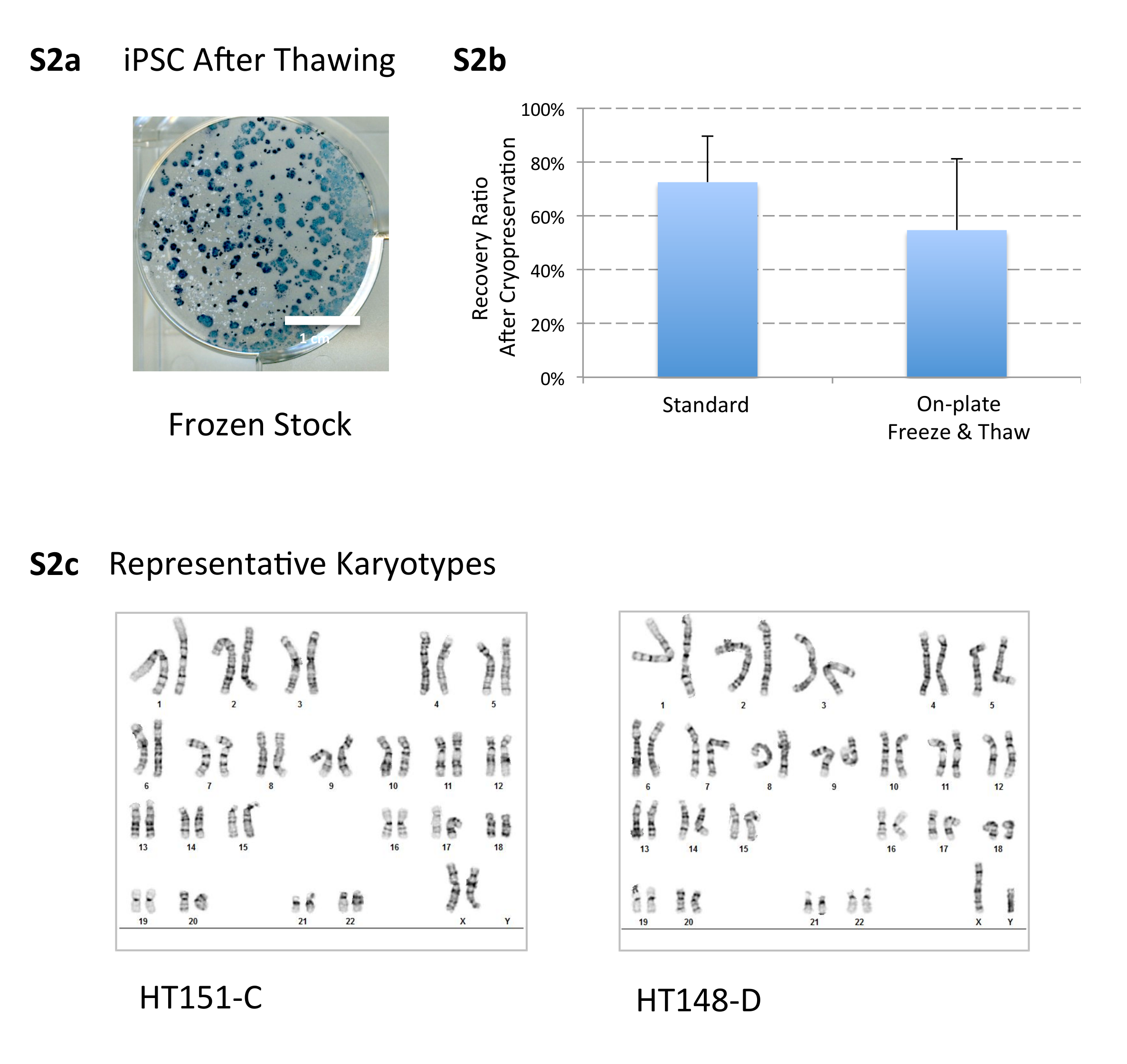
Figure S2. Cryopreservation during reprogramming process**

S2A. *The cryopreservation and recovery of the reprogramming stock.* CytoTune^TM^ 2.0-transduced fibroblasts were preserved 4 days after transduction, and later recovered and reprogrammed in E8-based medium. The plate was stained for APS 20 days after recovery. B. *Comparison of iPSCs against standard vial preservation.* Identical sets of H1 ESCs were prepared for cryopreservation in either cryovials or directly on the plate, and cells were then recovered and counted by flow cytometry. Experiments were done in triplicate (p>0.08) C. *Karyotypes from iPSCs recovered from cryopreservation on plate.*

**Supplementary Methods**

**Reprogramming with Sendai Virus in E8 based medium**

*Part 1:Transduction of human fibroblasts. Timing: 4-5 Days*

Thaw a low-passage-number of mycoplasma-free fibroblast culture and plate it in one well of a six-well dish in fibroblast medium. Incubate the cells, feeding every other day, until cells are 100% confluent. Once cells are confluent, passage cells on Day 1 into two 48 well plates. Every confluent well in a 6 well can be passaged into 1 well in each 48 well plate (2 plates total). Passage cells using TrypLE, and plate the cells in the two 48 well plates in 300 ul/well of fibroblast culture medium. Transfer the cells to a 37^o^C CO2 incubator for 3 hours.

After 2 hours, prepare the CytoTune^TM^ Sendai kit as per instructions, mixing the 4 viruses together. Add this virus preparation to the fibroblast medium, enough for 300 ul/well of the transduction mixture. Mix in polybrene (0.30 ul/well to be transduced, of a 1000X stock). Remove the medium currently on the cells, and add 300 ul/well of the Sendai virus/polybrene mixture. Incubate overnight at 37^o^C in a CO2 incubator.

*Part 2: Reprogramming. Timing:3-4 weeks*

On Days 1 and 3, replace the medium with fresh fibroblast medium. On Day 5, passage the well of reprogramming cells using TrypLE. TrypLE dissociation preserves cells better than regular trypsin-EDTA, and it does not require serum-based neutralization. To do this, remove the medium from the cells and rinse with 250 μl of EDTA dissociation solution. Remove the EDTA solution immediately and add 0.1 ml of TrypLE to the well. Incubate for 5 min in an incubator, and then wash the cells off the plate with 0.2 ml 10% albumin in PBS to inactivate TrypLE. For plating on 6 well plates:

Dilute the resuspended cells in 24 ml E8-based reprogramming medium containing Butyrate. Remove the Matrigel/DMEM-F12 used to coat two 6-well plates and add 2 ml of cells per well. This gives a final passage of one well of infected cells into two six-well plates. This is the preferred method if colonies will be picked manually.

For a high throughput method, the cells can be reususpended in 500 μl of E8-based reprogramming medium containing Butyrate and plated on 1 48-well plate coated with Matrigel. This method can be used if colonies are to be passaged by the EDTA method when mature. 48 well plates are coated with Matrigel in E8-based reprogramming medium containing Butyrate with a volume of 450 μl in the top row and 250 μl in the remaining rows. 50 μl of cells are placed in every well on the top row. After mixing, 50 μl of each well are transferred to each of the 3 wells below it. 5 μl of the top well are then transferred to each of the two bottom wells:

Top row: 1:10 (5x10^3^ cells), next 3 rows 1:100 (5x10^2^ cells), last 2 rows 1:1000 (5x10^1^ cells). The remaining 100 μl of cells can be plated on 2 wells of a 6 well plate as a backup or frozen as a D5 reprogramming stock.

Maintain the cells in E8-based reprogramming medium containing Butyrate, replacing medium every other day until day 20.

After day 20, begin feeding complete E8 media, replacing the medium every day.

Monitor the cells daily, if cells become too confluent while you are waiting for iPSC colonies to mature, cells may need to be passaged with EDTA at some point during the 2 weeks. Use the EDTA passaging method as described.

Twenty to thirty days after transduction, colonies should be ready for mechanical picking from a 6 well plate or, if single colonies have appeared in the wells of the 48 well plates, the well can be passaged by the PBS/EDTA method directly into a Matrigel-coated 12 well plate.

***Cryopreservation of iPS reprogramming on 48 well plates***

Remove media from the cells and add PBS/EDTA to well one time (no washing) After 5 minutes at room temperature, remove the PBS/EDTA and resuspend the cells in 150 μl of cryopreservation media (E8+ROCK inhibitor+ 10% DMSO). Let the plates sit on ice for 5 minutes, then place the plate between 2 pieces of Styrofoam and place in a larger Styrofoam box, and transfer the box to a -80^o^C freezer to store.

To thaw these cells, remove the plate from the -80 and place on top of a 37^o^C heat plate if available. Add 750 μl of warmed E8+ROCKi medium to each well and transfer to a 37^o^C incubator. After 30 minutes, check for cell attachment on plate, and remove the media, replacing with 500 μl of fresh warm E8+ROCKi medium and return to the 37^o^C incubator.

**qRTPCR in Fluidigm**:

RNA was purified using TRI Reagent® Solution according to Ambion's protocol. Residual DNA was removed using the TURBO DNA-free™ kit. Reverse transcription was carried out with Maxima H Minus Reverse Transcriptase (Thermo Scientific) primed with Poly N15-mer (Eurofins) with the recommended protocol. Prior to PCR, RNA template was removed with addition of Ambion Ribonuclease H, from E. coli. A Fluidigm 96x96 Dynamic Array chip was run with Qiagen Stem Cell Transcription Factors PCR Array primers, using the Fuidigm protocol for Fast Gene Expression Analysis using EvaGreen® on the BioMark System. Analyses and plots for RTPCR were generated using R 3.1.1 ^1^ with the data.table ^2^ and Bioconductor's ^3^ HTqPCR ^4^ packages. Ct normalization was done with the normalizeCtData function using options norm.rankinvariant, pseudo.median, and Ct.max value of 35. The heatmap was generated using the plotCtHeatmap function with euclidean distance clustering dendrograms.

**Reference**

1. R Core Team (2014). R: A language and environment for statistical computing. R Foundation for Statistical Computing, Vienna, Austria. URL [http://www.R-project.org/](https://mail.nih.gov/owa/redir.aspx?C=WMb4rTuTwEGz5Gq8g_J2cYlPLSasu9EIymaWwiq4wojWdPbtOnLh0TgXdHJrf4GpyUNXcHEeW3Q.&URL=http%3A%2F%2Fwww.r-project.org%2F).
2. M Dowle, T Short, S Lianoglou, A Srinivasan with contributions from R Saporta and E Antonyan. (2014). data.table: Extension of data.frame. R package version.  [http://CRAN.R-project.org/package=data.table](https://mail.nih.gov/owa/redir.aspx?C=WMb4rTuTwEGz5Gq8g_J2cYlPLSasu9EIymaWwiq4wojWdPbtOnLh0TgXdHJrf4GpyUNXcHEeW3Q.&URL=http%3A%2F%2Fcran.r-project.org%2Fpackage%3Ddata.table).
3. Heidi Dvinge and Paul Bertone (2009) HTqPCR: High-throughput analysis and visualization of quantitative real-time PCR data in R. Bioinformatics, 25(24):3325.
4. Bioconductor: Open software development for computational biology and bioinformatics R. Gentleman, V. J. Carey, D. M. Bates, B.Bolstad, M. Dettling, S. Dudoit, B. Ellis, L. Gautier, Y. Ge, and others 2004, Genome Biology, Vol. 5, R80.
